# Vaccination of lambs against *Haemonchus contortus* with the recombinant rHc23. Effect of adjuvant and antigen dose

**DOI:** 10.1371/journal.pone.0193118

**Published:** 2018-03-07

**Authors:** María Elena González-Sánchez, Montserrat Cuquerella, José M. Alunda

**Affiliations:** Department of Animal Health, Faculty of Veterinary Medicine, Universidad Complutense, Madrid, Spain; Instituto Butantan, BRAZIL

## Abstract

*Haemonchus contortus* is the most pathogenic gastrointestinal helminth of small ruminants. Natural or experimental repeated infections and several native antigens confer a partially protective immune response but vaccination with subunit antigens has been elusive. Promising results have been obtained with a recombinant form of a somatic antigen (rHc23). In this paper we present the results obtained in vaccination trials in lambs using two dosages of rHc23 and standard adjuvants. Six-months old Manchego females lambs were vaccinated with rHc23 (50 or 200 μg/dose) adjuvanted with 1mL aluminum hydroxide on days -42, -28 and -14 or with 200 μg/dose rHc23 and 5 mg Quil A on days -49, -28 and -7. Control lambs were kept receiving only the adjuvants the same days or no treatment. Moreover one group did not receive any treatment or infection. On day 0 vaccinated lambs, untreated animals and those receiving the adjuvant were infected per os with a monospecific single infection of 4000 L3 of *H*. *contortus*. Infection was kept for 58 days and follow-up included the determination of serum specific antibody response (ELISA, WB), hematological parameters (eosinophil counts, hematocrit) and fecal egg counts (epg). Absence of hematocrit alterations, reduction of helminth’s eggs output and abomasal parasite burden at the end of the experiment were the efficacy criteria of vaccination with the recombinant. Immunization with both adjuvants and antigen dosages elicited strong antibody responses particularly with Quil A. Vaccinated groups showed significant reduction of fecal egg excretion and abomasal helminth burdens. Highest protection of lambs against challenge was achieved with aluminum hydroxide and 200 μg/dose rHc23 with a reduction of over 70% of the abomasal burden and over 80% of fecal egg output. Results suggest that rHc23 could be a valuable recombinant candidate for vaccination against haemonchosis. No clear relationship was found between antibody levels and protection this pointing towards involvement of both humoral and cellular components in the protective response elicited by rHc23.

## Introduction

Infections of ruminants by gastrointestinal nematodes (GIN) are present worldwide. Among them haemonchosis, the disease caused by *Haemonchus contortus*, is probably the most relevant and represents ca.15% of all gastrointestinal diseases of small ruminants (http://www.fao.org). Infection by this gastric nematode, although present in all areas with significant sheep and goats numbers, is more common in warm areas and this helminth has been considered the “nemesis” of small ruminant production systems in tropical and subtropical regions of the world [1]. Distribution of this parasite relates to its ability to adapt to very different environmental conditions, high fertility of female worms and the peculiar relationship with the immune system of infected animals. Given the economic impact of the disease and its extension control of haemonchosis is a “must” for sheep and goat farmers. Anthelmintics, including their massive administration, have been the main tool to limit the infection. However, drug resistance of GIN is global [2,3] and costs associated to repeated medication of animals are hardly affordable for many farmers. Surely the effective control of haemonchosis will require an integrated approach using different control systems (e.g. anthelmintics, pasture rotation) of variable relevance depending on the exploited areas and animal breeds, management systems and production purposes. Among these systems immunoprophylaxis could be a useful approach. It is well known that under natural or experimental conditions sheep and goats develop resistance to reinfection after a primary exposition to the parasite [4–6]. Moreover, under experimental conditions, vaccination with different larval (L3) and adult antigens of *H*. *contortus* induces significant levels of immune protection [7–9]. Actually, using a similar approach to that used to develop Bm86 vaccination against ticks [10], a native antigen of *H*. *contortus* has been marketed (Barbervax®) and is widely used in Australia. However, this approach although valuable has several limitations including the required number of boosters (5–6) [1, 11], the limited protection elicited in some cases [12] and the ethical concerns on the use of infected animals to obtain the “native” antigen for immunization. The development of subunit helminth parasite vaccines for practical application would be a ground-breaking step in the control of GIN infections and in particular in haemonchosis [13]. Unfortunately none of the recombinant counterparts of the native antigens inducing protection, including H-gal-GP, has shown to elicit significant protection levels against *H*. *contortus* challenge [7, 9, 14, 15]. Standard vaccination of lambs with a naturally exposed antigen of adult *H*. *contortus* (Hc23) and a recombinant version, rHc23, has shown to elicit 70–80% reductions of fecal egg excretion and abomasal helminth burdens after experimental challenge [16,17]. Use of a recombinant antigen for vaccination against haemonchosis has important advantages (e.g. standardized production, easy handling and storage, ethical approach). Since Hc23 is a naturally exposed antigen, an efficacy over 60% would be considered as a control system [18]. Efficacy of vaccinations strongly depends, among other factors, on the antigen doses administered and the adequate adjuvant employed. In the present study we present the results obtained in vaccination trials against lamb haemonchosis with rHc23 with different antigen doses and adjuvants.

## Material and methods

### Adult soluble extract of *H*. *contortus*

Adult *H*. *contortus*, largely females, from the abomasa of monospecifically infected donor lambs, were extensively washed in PBS, treated with proteases inhibitors (Roche, Mannheim) and stored at -80°C. Thawed helminths were subjected to 8 freezing-and-thawing 20 min cycles, homogenized in a glass-in-glass tissue homogenizer and centrifuged at 30000xg, 30 min, 4°C. Supernatants (Adult soluble extract, ASE) were recovered, protein concentration determined with RC/DC protein assay (Bio Rad) and stored at -80°C until used.

### Production of recombinant protein rHc23

Recombinant rHc23 was obtained in our laboratory using the insert from the expression vector pET29b in competent *Escherichia coli* XL2-blue frozen cells [16]. After thawing bacteria were grown in LB-kanamicine (Sigma) medium and the plasmid was purified with QIAprep Spin Miniprep kit (Qiagen) and transformed in *E*.*coli* BL21 (DE3). Protein was expressed with a final concentration of 0.5M isopropyl β-D-thiogalactopyranoside (IPTG) (Roche). Bacteria were lysed by sonication and the recombinant protein (rHc23) was purified by affinity chromatography in nickel column. Eluate was dialyzed against PBS, lyophilized and stored at -20°C until used. For lamb immunizations and immunological determinations the protein was resuspend in PBS, the protein contents estimated and adjusted to the required concentration.

### Parasites

Infective larvae (L3) were obtained from donor lambs with monospecific infections of *H*. *contortus*. This isolate was originally obtained from Merck, Sharp & Dohme Spain and has been kept by serial passage in lambs for over 25 years at our facilities. Fecal material was cultured (26°C, 80% relative humidity, 7 days) and L3 were recovered with a Baermann apparatus [19]. Cleaned larvae were maintained in tap water at 4°C until used.

### Lambs and experimental design

Forty five Manchego breed lambs were obtained from a local producer (Explotación El Navajo, Guadalajara) when they were 4 months old age. After arrival all lambs were treated with albenzadole (Albendex 10%, SP Veterinaria SA), subjected to quarantine and housed at the animal facilities (ES280790000137) of the Veterinary Faculty, UCM (Madrid) under conditions precluding undesired helminth infections. Animals were fed with commercial pelleted food (El Arca de Noé, Guadalajara, Spain) and hay and tap water ad libitum. Animals were divided in a stratified way (live weight) onto 7 groups. Groups 1 (G1) and 2 (G2) were immunized with rHc23 (50 or 200 μg/dose, respectively) and 1 mL aluminum hydroxide [Al(OH)_3_] (Brenntag) by intramuscular injection on days -42, -28 and -14 before challenge. Group 3 (G3) (adjuvant control group) was treated only with Al(OH)_3_. Lambs from Group 4 (G4) were immunized, by intramuscular injection, with 5 mg Quil A (Brenntag) and 200 μg/dose of rHc23 on days -49, -28 and -7 before challenge. Group 5 (G5) animals (adjuvant control group) only received Quil A on the same days. Group 6 (G6) lambs did not receive any treatment and group 7 (G7) was kept as untreated and uninfected control group. On day 0, animals from groups 1 to 6 were challenged with a monospecific infection of 4000 L3 of *H*. *contortus*. At the end of the experiment (58 days post infection, pi) all animals were sacrificed at a local slaughter house (Villarejo de Salvanés, Madrid), abomasa recovered and taken under refrigeration to the lab to estimate the adult parasite burden. Details concerning animal numbers and experimental design are given in Table 1. Experimental design and procedures were approved by the Ethical Committee of the Universidad Complutense and authorized by Regional Government (Comunidad de Madrid) (PROEX 75/14). To avoid the unnecessary use of animals control groups (G6 and G7) were used simultaneously for other simultaneous experiments.

**Table 1:** Experimental design of the vaccination trial of lambs vaccinated with rHc23 against *Haemonchus contortus* challenge.

	Al(OH)_3_	Al(OH)_3_ and rHc23	Quil A	Quil A and rHc23	*H*. *contortus* (4000 L3)
**Group 1 (7)**		**+** (3x50μg rHc23)			+
**Group 2 (7)**		**+** (3x200μg rHc23)			+
**Group 3 (6)**	+				+
**Group 4 (7)**				**+** (3x200μg rHc23)	+
**Group 5 (6)**			+		+
**Group 6 (7)**					+
**Group 7 (5)**					-

In brackets: the number of animals from each experimental group.

**+:** Treatments received

### Follow up, end point and efficacy criteria

#### Blood sampling

Blood samples were obtained by jugular venipuncture in evacuated tubes (Vacutainer) on days -49, -21, 0, 7, 14, 21, 28, 35, 42 and 49 pi. Samples for serum analyses were allowed to clot, centrifuged at 800 xg and stored at -20°C until use. Packed cell volume (PCV) and eosinophil counts were performed using standard laboratory techniques with blood samples with EDTA.

#### Enzyme linked immunosorbent assay (ELISA) and Western blotting

ELISA and Western blotting were carried out with a previously published method [20] with slight modifications. ELISA conditions were determined in a checkerboard manner. Briefly, 96 well microplates (Nunc) were coated with 100 μL of ASE (5μg/mL) or rHc23 (1μg/mL) at 4°C for 16 h. Plates were blocked with 5% bovine serum albumin (BSA) (1 h, 37°C). Individual sera were used at a 1/200 dilution in PBS-Tween for 1 h at 37°C. Conjugate (alkaline phosphatase-labeled rabbit anti-sheep IgG, Sigma) was diluted 1/8000 for ASE-coated plates and 1/32000 for rHc23-coated wells and incubated for 1 h at 37°C. Substrate was 1 mg/mL 4-nitrophenil phosphate (Sigma) and optical density (OD) was read with a Multiskan GO (Thermo Fisher Scientific) at 405 nm.

Electrophoresis of ASE of *H*. *contortus* (14.7 μg/cm of gel) and rHc23 (2.65 μg/cm of gel) was carried out with a 12.5% acrylamide-bisacrylamide (Merck) gel. Molecular mass markers (MW) were from GE Healthcare (UK) and electrophoresis was run at 80 V for 30 min followed by 1 h at 150 V. Electropherogram was transferred to Immobilon P membranes (Millipore), for 2 ½ h, 400 mA. Membranes were washed and blocked for 1 h with 5% skimmed milk (Sveltesse, Nestlé), cut onto strips (3 mm width) and incubated with pooled sera (1/100 dilution in TBS-5% skimmed milk) from each group corresponding to the 21 days pi sampling for 3h, shaking, at 37°C. Conjugate (anti-sheep IgG-HRP, Sigma) in TBS-T was employed at a 1/1000 dilution for 1 h at 37°C. After washing (TBS-T, TBS) substrate was added (84 mL TBS + 0.05% hydrogen peroxide + 0.5 mg/mL 4-chloro-1-naftol+ 16.8 mL methanol) and the reaction stopped by rinsing in distilled water.

#### Parasitological determinations

Individual fecal samples were taken from the rectum weekly from the beginning of the patency until the week 7 pi. Fecal samples were analyzed with a modified McMaster technique [19] and results given as eggs per gram of feces (epg). Both arithmetic and geometric mean egg counts were determined for each sampling point. Individual counts of epg were log (x+1) transformed for normalization. Cumulative fecal egg output was estimated using the trapezoidal method to determine areas under the curve (AUC) of the animals and groups. Abomasa from the experimental animals were opened by the major curvature and a 10% of abomasal contents were preserved with 5% formaline at RT until counting of adult helminths.

#### Efficacy criteria

Successful immunization of the experimental animals was determined by serum anti-rHc23 antibody levels. Reduction of fecal egg output, abomasal parasite burden and hematocrit were considered as estimates of the level of protection achieved after immunization with the recombinant rHc23.

#### Statistical analysis

Values given are mean ± standard deviation. Repeated measures (ELISA, epg counts and PCV values) were analyzed with a linear mixed model followed by a post hoc test (Tamhane multiple comparison for ELISA and Bonferroni for epg and PCV) between experimental groups. Homogeneity of groups was tested by Tukey test. Cumulative fecal egg output (AUC), eosinophil counts and abomasal helminth burdens were analyzed by 1-way ANOVA. In all cases significance was set at *P*<0.05.

## Results

### Serum antibody response

**Fig 1:**
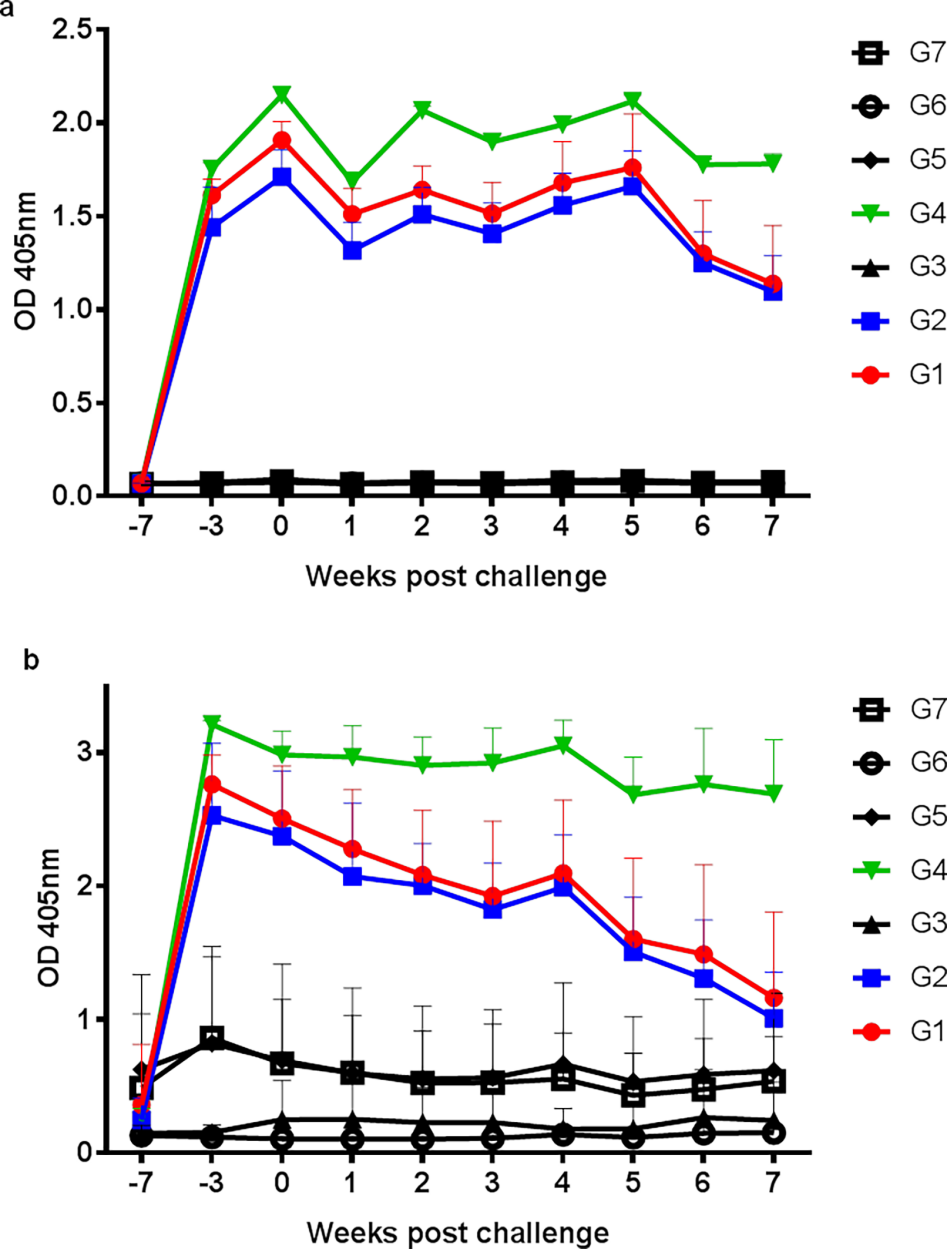
Serum specific IgG response of vaccinated lambs with rHc23 after challenge with 4000 L3 *Haemonchus contortus*. Fig 1A: Anti-rHc23 serum IgG response of experimental lambs. Fig 1B: Anti-Adult Soluble Extract of *H*. *contortus* (ASE) serum IgG levels along the experiment. Sera were diluted 1/200 and data are mean±standard deviation of the optical density (OD) values found before infection (weeks -7 and -3), the day of the infection (week 0) and weekly afterwards up to week 7 post challenge.

Immunization with rHc23 with both adjuvants (aluminum hydroxide and Quil A) elicited a strong IgG antibody (Ab) specific response against the recombinant in the three vaccinated groups (G1, G2, G4) (Fig 1A).There were time-related differences between groups from the second injection (week -3) until the end of the experiment. Comparison at each sampling time showed that vaccinated lambs had significantly higher Ab levels (*P*<0.05) than all other groups, particularly those immunized with the recombinant and Quil A.

Serum IgG response against ASE (Fig 1B) showed a comparable pattern. The three immunized groups strongly recognized the soluble extract of *H*. *contortus*. Lambs vaccinated with Quil A (G4) were significantly different to non-vaccinated animals during all the experimental period. By its part, animals immunized with the recombinant + Al(OH)_3_ (G1, G2) showed a less sustained Ab response to ASE and no differences with unvaccinated animals were present from week 7 pi onwards. Primary infection without any adjuvant (G6) did not elicit any significant response against *H*. *contortus*. Higher OD average values were found in the groups only receiving the adjuvants (G3 and G5) although there were not differences with primarily infected (G6) or uninfected control lambs (G7).

**Fig 2:**
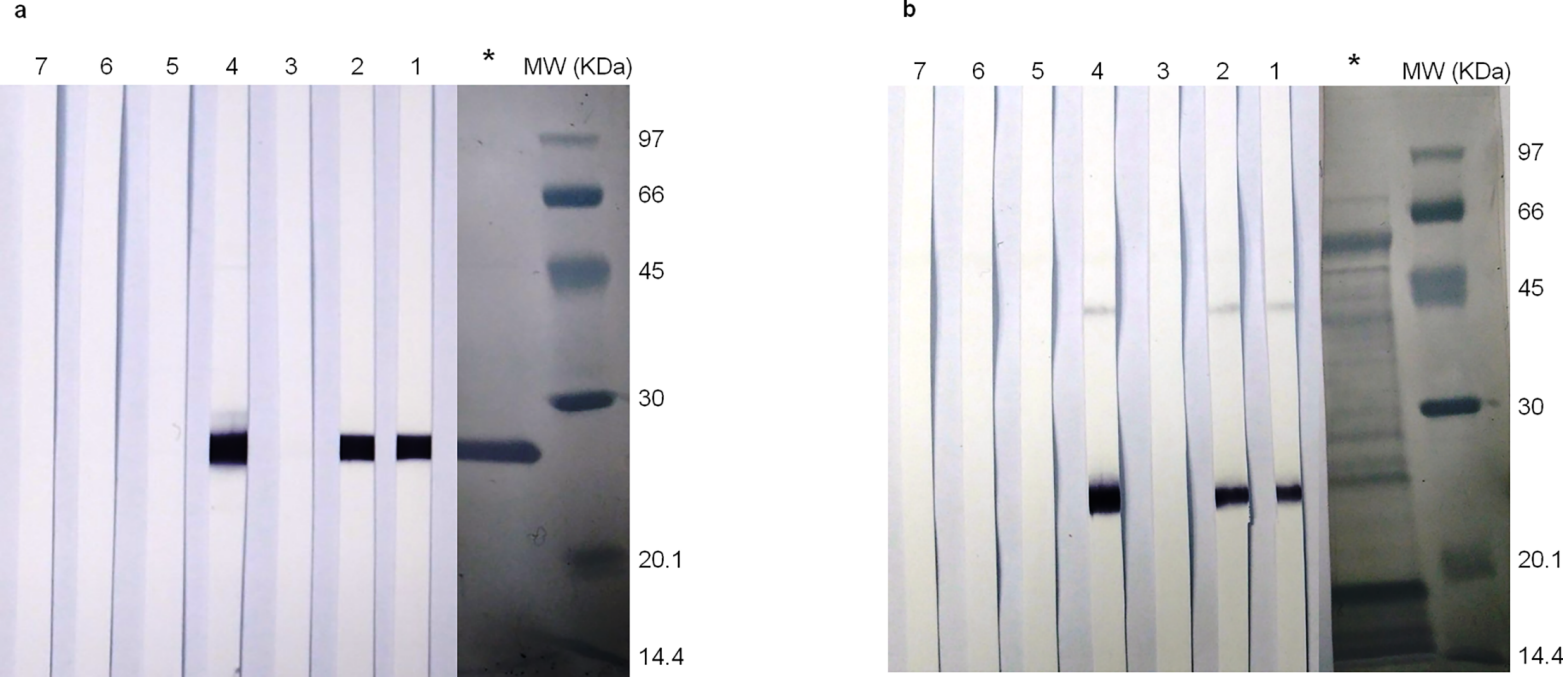
Immune recognition of rHc23 and Adult soluble extract (ASE) by lambs vaccinated with rHc23. Fig 2A: Immune recognition of rHc23 (2.65 μg/cm of gel) by pooled sera from the experimental groups of vaccinated lambs on week 3 post challenge. Fig 2B: Immune recognition of ASE (14.7 μg/cm of gel) by pooled sera from the experimental groups of vaccinated lambs on week 3 post challenge. Proteins were separated by SDS-PAGE and pooled sera were 1/100 diluted. Lane 1: G1; lane 2:G2; lane 3: G3; lane 4: G4; lane 5:G5; lane 6: G6; lane 7: G7. MW: molecular weight markers. *: Amido black stain of membrane with the transferred protein. See Table 1 for details of the groups.

Results obtained in the ELISA showed that immunization was successful and high anti-rHc23 Ab levels were produced irrespective of the adjuvant and the antigen amount employed with the aluminum vaccination. Comparable antibody responses using rHc23 or ASE, particularly with the saponin, were found by ELISA and this as confirmed by WB since pooled sera from immunized groups reacted with rHc23 (Fig 2A) and also with ASE of *H*. *contortus* (Fig 2B). Reactivity of Quil A-treated group (G4) was apparently more evident. It is noteworthy to indicate that in the WB with ASE besides the reactivity at 23 KDa there was a faint reaction ca. 46 KDa.

### Eosinophil counts and Packed cell volume (PCV)

All lamb groups showed an elevation in eosinophil counts along the experiment as assessed by the differences between day 0 and day 42 pi. There were some intergroup variations (*P*<0.05) in the last count although all values were within the normal physiological range. Despite the infection-related reduction in PCV observed after challenge, values were within the physiological range and no evidence of anemia was observed.

### Fecal egg output

**Fig 3:**
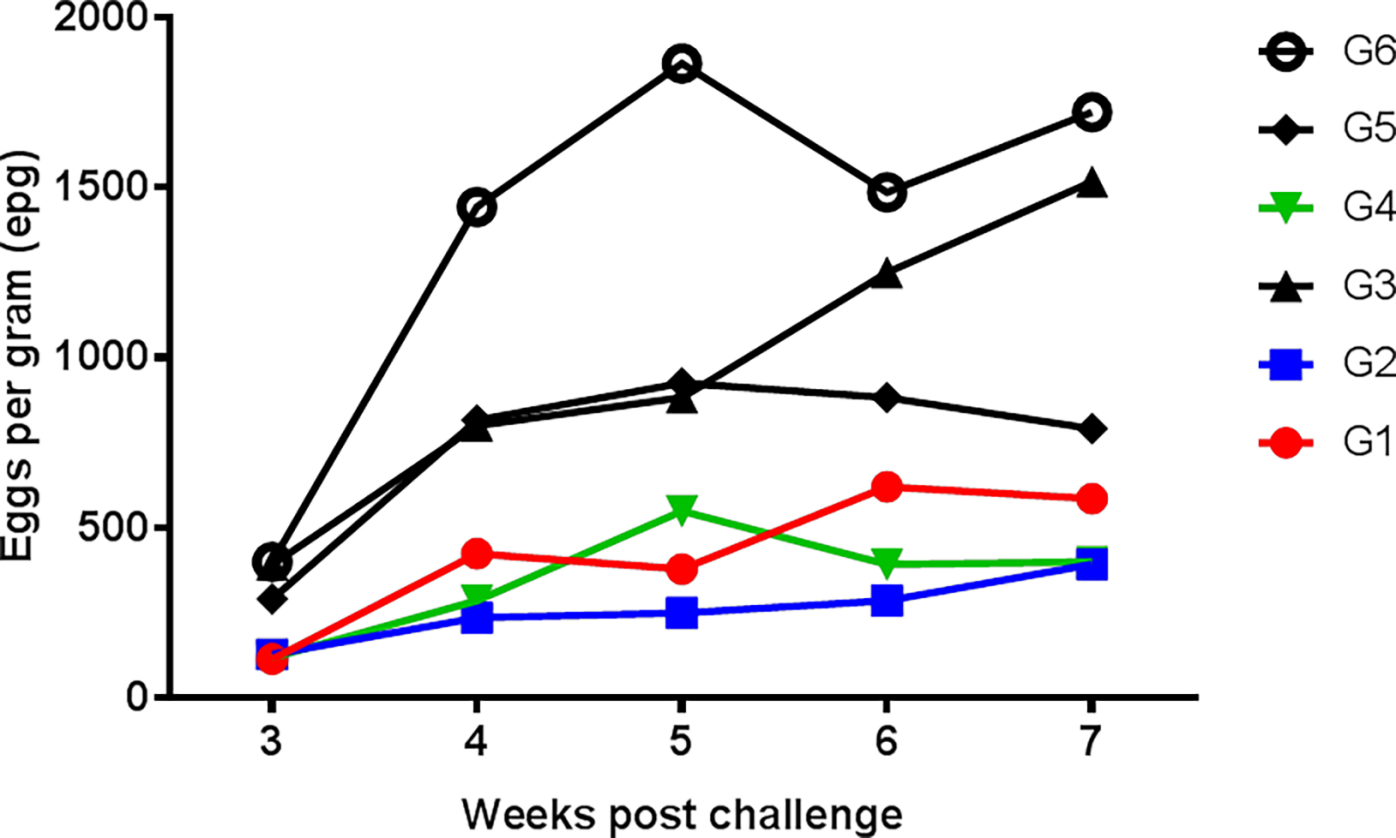
Fecal egg output of vaccinated lambs with rHc23 after challenge. Values are geometric means of individual log transformed (log x+1) *Haemonchus contortus* eggs excretion (eggs per gram, epg) from week 3 post challenge onwards. Individual fecal analyses were performed with a modified McMaster technique. Symbols as in Fig 1.

Fig 3 shows the fecal output of *H*. *contortus* eggs (epg) along the experiment and Table 2 presents the cumulative fecal egg counts (AUC) of the experimental groups. There was a high intergroup variation in the challenged lambs. Repeated measures analysis showed significant (*P*<0.05) effects of both time of infection and lamb group. All vaccinated groups (G1, G2 and G4) shed lower number of eggs on weeks 4, 5, 6 and 7 pi (*P*<0.05) than unvaccinated and challenged animals. Despite of individual variations within each animal group, at each sampling time and along the experiment, administration of adjuvant alone (G3, G5) did not elicit any reduction of epg values from week 4 pi onwards compared to the unvaccinated lambs (*P*>0.05). Pattern of *Haemonchus* eggs shedding of vaccinated animals were comparable without any apparent effect of antigen dose administered (G1 vs G2) or adjuvant employed (G2: alum vs. G4: Quil A).

**Table 2:** Cumulative fecal *Haemonchus contortus* eggs output (AUC 21–49 dpi) of lambs vaccinated with rHc23.

	AUC ± sd
Group 1	12415 ± 7123.79 ^[^^6^^]^
Group 2	7225 ± 2449.4 ^[^^3^^,^ ^6^^]^
Group 3	27212.5 ± 13783.4 ^[^^2^^]^
Group 4	10415 ± 4484.87 ^[^^6^^]^
Group 5	22166.67 ± 9541.38
Group 6	40975 ± 21142.78 ^[^^1^^,^ ^2^^,^ ^4^^]^

Data are mean

± standard deviation, sd.

Values with different superscripts indicate significant differences (*P*<0.05) between groups.

Considering the cumulative FEC, vaccinated animals (G1, G2, G4) had significantly lower AUC values than unvaccinated and challenged lambs (Table 2). The lowest elimination was found in the groups of lambs vaccinated with the highest dosage of recombinant and aluminum as adjuvant (G2). In this group vaccination elicited a reduction of eggs eliminated over 80% (82.37% ±5.98%) and slightly lower in Quil A-adjuvanted group (G5) (74.58%±10.94%) and the animal with the low recombinant dose (G1) (69.7±17.39%). Statistical analysis did not show any significant effect of antigen dose or adjuvant employed (*P*>0.05). However it should be noted that reduction found in G2 lambs was not related to the effect of Al(OH)_3_ alone (G3) (*P*<0.05) whereas protection found in the animals vaccinated with the recombinant and Quil A (G4) was not significantly different to the egg output reduction observed in the lambs treated only with the saponin (G5) (*P*>0.05).

### Abomasal helminth burden

Wide intragroup variations in abomasal helmith burdens were found (Table 3) and the number of adult *H*. *contortus* was low representing a 7.44% establishment rate in the infected+untreated animals (G6). Higher numbers of females were recovered in all groups except the infected non vaccinated animals. Thus, female/male ratio ranged from 1.61 (G2) and 0.79 (G6). Immunization with rHc23 with both adjuvants elicited a significant (*P*<0.05) reduction of adult parasite burden compared to the infectedandunvaccinated lambs (G6). Average reduction ranged from 47.3 ±35.4% in G4 (Quil A) to 71±14.3% in G2 [Al(OH)_3_]. No significant effect of antigen dose was found with aluminum adjuvant (G1 vs G2). On average lambs receiving the adjuvant alone had lower parasite burdens although differences were not significant. However it is worth pointing out that the lowest helminth burden was observed in the group vaccinated with the highest Ag dose and alum. This group was significantly different (*P*<0.05) to both adjuvant control group (G3) and the animalsvaccinated with Quil A (G4).

**Table 3:** Abomasal adult *Haemonchus contortus* burdens (males, females and total) of vaccinated lambs with rHc23.

	Adult males	Adult females	Total helminths	Reduction (%)
**Group 1**	61.29±38.9	85.71±53.5	147± 91.1 ^**[**^^**6**^^**]**^	50.6±30.6
**Group 2**	33±19.64	53.29±25.6	86.29±47.2 ^**[**^^**3**^^,^^**5**^^,^^**6**^^**]**^	71.0±14.3
**Group 3**	96.5 ±72.3	123.8 ± 65.1	220.3±137.1^**[**^^**2**^^**]**^	26.0±46.0
**Group 4**	66.43±49.3	90.4±57	156.9±105.5 ^**[**^^**6**^^**]**^	47.3±35.4
**Group 5**	102.5±60.7	125±69.6	227.5±129^**[**^^**2**^^**]**^	23.6±43.3
**Group 6**	167.2±79	130.7±87.2	297.8±160.2^**[**^^**1**^^,^^**2**^^,^^**4**^^**]**^	————-

Data are mean

± standard deviation.

Only lambs receiving challenge infection (4000 L3, single dose) are included.

Values with different superscripts indicate significant differences (*P*<0.05) between groups.

## Discussion

Despite the evidence of the capacity of small ruminants to mount a partially protective response after natural or experimental infections of *H*. *contortus*, and the advantages of the vaccination with a recombinant product progress has been modest. A recombinant *H*. *contortus* antigen (rHc23) has shown to confer over 80% protection (eggs excretion, helminth burden) against challenge in lambs using aluminum hydroxide as adjuvant and a standard immunization schedule with three injections of recombinant (100μg each) [17]. Efficacy of vaccinations strongly relies on the adequate selection of adjuvant to elicit the effective immune response [21]. Moreover, antigen dose administered is important on economic grounds, and dose-response variations have been reported in other host-pathogen systems [22–25] Results showed that immunization of lambs using both adjuvants (Quil and aluminum) elicited high levels of circulating anti-rHc23 *Haemonchus* IgG. Higher and more sustained ELISA response against rHc23 and ASE in Quil Aand rHc23 is consistent with the double activation of the immune system by the saponin (Th1, Th2) [26, 27] and the rapid decline of aluminum-adjuvanted antigens in absence of repeated injections [21]. Lamb serum reactivity of the three vaccinated groups against the recombinant and ASE, and the comparable WB recognition patterns, confirmed the similarity of the recombinant and the native antigen present in adult *H*. *contortus*. Two of the lamb groups (G5, G7) displayed some unspecific reactivity in ELISA with ASE. The possibility of some cross reactivity of the recombinant with other unrelated antigens cannot be ruled out. However, no recognition was observed in WB and no *H*. *contortus* infection was present in the supplier farm. Absence of detectable specific anti-*Haemonchus* Ab after challenge is consistent with previous observations in experimental primary infections of lambs [16]. Eosinophils have been incriminated in the killing of *H*. *contortus* larvae [28]. In the present experiment, there was an elevation of peripheral eosinophil counts in all vaccinated lambs with rHc23 although numbers were within the physiological range and no correlation was found between their numbers and the key parameters considered as markers of protection.

As efficacy criteria of the vaccination trial with rHc23 parasitological parameters in vivo and postmen (epg, abomasal helmint burden) and PCV variations were considered. Infective dose (4000 L3) could account for the lack of significant effects of infection on PCV.

Fecal egg output is a valuable *in vivo* phenotypic marker of resistance in haemonchosis [29]. By its part abomasal parasite burden besides its value as marker of effective protection elicited [30, 31] is the actual responsible of pathogenesis in haemonchosis. Despite the expected individual variations within each group vaccinated lambs (G1,G2, G4) shed significantly **(***P*<0.05) lower numbers of *Haemonchus* eggs along the experiment from the week 4 until the end. Protection elicited by vaccination was dependent on the antigen administered rHc23 since no differences were found in any of the analysis between untreated and infected control animals and the lambs receiving only the adjuvants. Estimation of epg values is subjected to a number of potential biases (e.g. degree of humidity of fecal material, daily variations). Thus, a better estimate of egg production would be the cumulative fecal egg count. Results confirmed the significant effect of vaccination, with both adjuvants and antigen dose, on the reduction of eggs shed to the environment by challenged animals. Reduction found in aluminum adjuvanted high antigen dosage of the recombinant (82.3%) was similar to that previously found with native protein [16], the recombinant rHc23 using the same adjuvant in Assaf lambs [17] and slightly lower than those recently obtained in Baladi breed sheep with this recombinant [32]. Antigen dose (G1 vs G2) apparently had no effect on epg counts (*P*>0.05) but the reduction elicited by vaccination with alum adjuvant was superior to that achieved with Quil A (*P*<0.05). The non significant reduction of cumulative epg found in lambs receiving only adjuvant could be related to the unspecific protection elicited as has been reported for Quil A [26].

Abomasal helminth burden is probably, along helminth size and fertility, one of the most relevant parameters to assess the efficacy of vaccination against *H*. *contortus*. Establishment rate found was very close to the values obtained with this helminth isolate in experimental infections and vaccination trials [17, 33]. Results showed that all vaccinated groups with rHc23 had significantly lower numbers of abomasal helminths this supporting the protective effect of the recombinant. Higher protection was observed with the higher antigen dose and aluminum hydroxide as adjuvant compared to the vaccination with rHc23andQuil A. Despite the limited number of animals our results support the superior value of Al(OH)_3_ as adjuvant in rHc23 vaccination against lamb haemonchosis. Lack of significant differences between the lambs vaccinated with the saponin and recombinant and the group receiving only the adjuvant suggests that, in our conditions, Quil A behaved as immunomodulator and near 50% of protection elicited in vaccinated lambs was apparently due the unspecific protection provoked by the saponin alone. On the contrary, aluminum alone did not show any relevant effect.

There was an apparent tendency towards higher protection achieved (epg, helminth burden) with higher amount of antigen but the differences were not significant and this could be related to the frequent presence of responder and non responder lambs within a given sheep group. It is considered that natural and acquired immune resistance to gastrointestinal nematodes of sheep is associated to a predominant Th2 response [5, 6, 34]. Probably both cellular and humoral responses are involved in the protection by rHc23 since higher levels of Ab elicited and maintained by Quil A did not correlate to higher protection of lambs. This lack of correlation has also found in vaccination trials with a larval surface antigen of this nematode [35, 36].

Infections by *Haemonchus* are widespread and reports of anthelmintic resistance in small ruminants are growing [3,37]. Vaccination could have important effects on farming sustainability, reduction of environmental residues and the presence of pharmacologically active drugs in meat and milk. By a number of reasons recombinant products would be an advantage. In this scenario, vaccination with the recombinant rHc23 has shown significant protection levels on major parameters (epg, abomasal helminth burden) (70%-over 80%) against haemonchosis. Obviously there is wide room for improvement (e.g. vaccination schedule; improvement of recombinant production) but combined results from this experiment and those obtained previously [17, 32] confirm the role of this recombinant as subunit vaccine candidate against *H*. *contortus* infection [13] and strongly support the trials under field conditions.
